# Synergistic effects of dietary RISCO-NUTRIFOUR probiotic on broiler performance, upregulation of intestinal immunoglobulin A and mucin-2 genes, enhancement of occludin expression, and downregulation of HSP70 under heat stress

**DOI:** 10.3389/fvets.2026.1776015

**Published:** 2026-04-22

**Authors:** Abdulaziz A. Al-Abdullatif, Maged A. Al-Garadi, Mohammed M. Qaid, Abdulkareem M. Matar, Mohsen M. Alobre, Mohammed A. Al-Badwi, Elsayed O. Hussein, Gamaleldin M. Suliman

**Affiliations:** Department of Animal Production, College of Food and Agriculture Sciences, King Saud University, Riyadh, Saudi Arabia

**Keywords:** broiler chickens, heat stress, HSP70 gene expression, probiotics supplementation, tight junction protein genes

## Abstract

Heat stress (HS) remains a major challenge in poultry production, negatively impacting performance, gut health, and immune function. This study investigated the synergistic effects of a novel probiotic solution, RISCO-NUTRIFOUR (RNFS), on growth performance, immune modulation, and intestinal barrier integrity in heat-stressed broilers. A total of 288 Ross 308 broiler chicks were allocated to six dietary treatments, including RNFS at three inclusion levels [0.4, 0.2, and 0.1% “(4, 2, and 1 L RNFS/ton feed)”], 0.1% *Bacillus subtilis*, 0.1% *Saccharomyces cerevisiae*, and a negative control. Over a 42-day trial, RNFS supplementation improved (*p* < 0.05) average daily weight gain (ADWG), feed conversion ratio (FCR), and intestinal gene expression of immunoglobulin A (IgA), occludin, and claudin-1, while downregulating the heat shock protein HSP70. These improvements exhibited dose-dependent patterns, with productive performance indices improving at lower RNFS doses, while intestinal gene expression was enhanced at higher inclusion levels. No adverse effects were observed on lymphoid organ development or core metabolic indicators. RNFS also modulated serum lipid profiles favorably and demonstrated a robust antioxidant and immunomodulatory phytochemical profile. These findings highlight RNFS as a promising probiotic feed additive to mitigate HS and enhance gut health and productive performance in broilers.

## 1 Introduction

Broiler chickens raised in tropical and subtropical climates are increasingly at risk of HS, a major environmental hazard that severely affects their health, productivity, and welfare (1, 2). Heat stress reduces feed intake and growth performance while disrupting metabolic, electrolyte, and endocrine functions in poultry (3–5). One of the most affected organs is the intestinal tract, which plays a central role-not only in nutrient absorption but also as a frontline barrier against pathogens and toxins. HS compromises intestinal integrity, leads to oxidative stress, and triggers inflammatory responses, ultimately affecting the health and performance of broiler chickens (6–8).

With the increasing effects of global warming, heat-related losses in poultry production are becoming more frequent and severe (9, 10). Broilers are particularly vulnerable as they cannot sweat and have a high metabolic rate (11, 12). Heat-induced oxidative stress leads to an overproduction of reactive oxygen species (ROS), which can damage DNA, mitochondria, and cell membranes, suppress immune responses, and promote chronic inflammation (13, 14). This cascade of physiological disorders contributes to increased morbidity, low weight gain, and even mortality (13, 15).

In response to these challenges, dietary interventions—particularly those containing probiotics—have shown promise as strategies to improve resilience to HS (16, 17). Traditionally, antimicrobial growth promoters (AGPs) have been included in poultry feed to maintain gut health and improve performance. However, due to growing concerns over antimicrobial resistance, the industry is shifting towards natural, safe, and sustainable alternatives (18). Probiotics—live microorganisms that have health benefits—are among the most promising substitutes for AGPs. Their effectiveness is based on their ability to restore microbial balance in the gut, strengthen the integrity of the epithelial barrier, stimulate the activity of digestive enzymes, and modulate immune responses (16, 19, 20).

Commonly used probiotic strains include *Bacillus* spp., *Lactobacillus* spp., *Bifidobacterium* spp. and live yeast *Saccharomyces cerevisiae*. These microorganisms have been shown to have the potential to reduce oxidative stress and support gut health (16, 19, 20). Recent research has also shown that they are able to modulate tight junction (TJ) proteins such as occludin, mucin-2 and immunoglobulin A, which are important for maintaining the intestinal barrier and immune surveillance (6–8, 21, 22).

Despite promising outcomes, many commercial probiotic products and research models are based on single-species or limited multi-species formulations (typically one or two dominant microbial taxa), which may restrict functional diversity compared with more compositionally complex consortia “RNFS.” There remains a gap in understanding the synergistic benefits of multi-strain and multi-species probiotics—particularly those combining bacteria and yeast organisms—for gut health and immune function under real-world HS conditions (20, 23). While previous studies have demonstrated the benefits of single- or dual-strain probiotics in mitigating HS effects, few complex, multi-strain formulations containing both bacteria and yeasts have been studied. Furthermore, there is limited data on how such mixtures affect molecular markers of gut barrier function and systemic stress in heat-stressed broilers (6, 23).

The current study investigates the application of RISCO–NUTRIFOUR^®^ Solution (RNFS), a novel multi-strain and multi-species probiotic containing *Bacillus subtilis, Saccharomyces cerevisiae, Lactobacillus parabuchneri*, *Lactobacillus harbinensis, Rhodopseudomonas palustris, Rhodopseudomonas shaeroides*, and *Candida ethanolic*. This formulation has been developed to provide enhanced antioxidant, anti-inflammatory, and gut-protective effects through potential synergistic microbial interactions, including cross-feeding and complementary metabolic functions among consortium members. For example, an inclusion of *Rhodopseudomonas* spp. and *Candida ethanolic* broadens the functional spectrum of the probiotic consortium, as *Rhodopseudomonas* contributes bioactive metabolites with antioxidant and metabolic-regulatory roles (24, 25), while *Candida ethanolic* provides enzymatic activities and supports microbial ecological balance, collectively enhancing gut stability and stress resilience (26, 27).

The study hypothesizes that RNFS supplementation will mitigate the detrimental effects of HS in broilers by improving growth performance, enhancing gut barrier function through upregulation of mucin-2, occluding, and IgA genes, and downregulating stress biomarkers such as HSP70. This study aimed to investigate the effects of multispecies RNFS probiotic supplementation as a natural and sustainable alternative to AGPs on broiler growth performance and feed efficiency under HS, compared with single-species probiotic treatments (*Bacillus subtilis* or *Saccharomyces cerevisiae*) used as positive controls, and a no-additive group serving as the negative control. In addition, the effect of the RNFS probiotic on gut integrity will be assessed by analyzing the expression of key intestinal tight junction and immune-related genes, including mucin-2, occludin, claudin-1, and IgA. The study also investigates the antioxidant and anti-stress properties of the RNFS probiotic by measuring the expression of HSP70, a biomarker for HS.

## 2 Materials and methods

### 2.1 Study location

This experiment was carried out at the Experimental Poultry Research Unit, Department of Animal Production, King Saud University (KSU), Riyadh, Saudi Arabia.

### 2.2 Birds, housing, and experimental design

A total of 288 1-day-old Ross 308 male broiler chicks, sourced from Al-Khumasia Hatchery, were used in this study. Chicks were individually weighed and randomly assigned to six treatment groups, each comprising 8 replicates with 6 birds/replicate. The dietary treatment groups were as follows:

*T1-T3*: Basal diet supplemented with RNFS probiotic at inclusion levels of 0.4% (4 L/ton feed), 0.2% (2 L/ton feed), and 0.1% (1 L/ton feed), respectively. Dosage selection was guided by manufacturer recommendations and prior pilot observations, and is consistent with previously reported inclusion ranges for multi-species RNFS supplementation in both water and feed (28). The proportional composition of the seven microbial strains in RNFS remained constant across inclusion levels; increasing dietary inclusion from 0.1 to 0.4% proportionally increased the CFU delivered per kg feed without altering strain ratios.

T4: Basal diet supplemented with 0.1% *Bacillus subtilis* powder (1 kg/ton feed).

T5: Basal diet supplemented with 0.1% *Saccharomyces cerevisiae* powder (1 kg/ton feed).

T6: Basal diet with no probiotic additive (negative control).

The single-probiotic treatments (T4: *Bacillus subtilis* PB6; CloSTAT^®^, 1:128, Kemin Industries, USA, and T5: *Saccharomyces cerevisiae*; SafMannan^®^, SafMannan, Phileo, Lesaffre, France) were included as industry-relevant comparators to differentiate the effects of the multi-species RNFS consortium from conventional single-species probiotic supplementation, serving as practical benchmarks rather than mechanistic equivalents.

The diets have been formulated in mash form according to the nutritional specifications of the ROSS 308 broiler management guidelines (Aviagen). Ingredient values and proximate chemical composition of each period are shown in Table 1. The birds had *ad libitum* access to water and feed during the 42-day trial. They were housed in battery cages with a controlled environment (58 × 50 × 35 cm) at a stocking density of 6 birds per 0.30 m^2^. The room temperature started at 35 °C on day 1 and was lowered by 0.5 °C daily until it reached 23 ± 1 °C on day 24 and remained constant at this level until day 28. The heat stress model imposed as environmentally cyclic heat stress rather than naturally ambient exposure. The finisher phase was selected because it represents the period of greatest metabolic heat production and highest susceptibility to heat load in broilers. For 29–42 days, the broilers were cyclically exposed to a high temperature (35 ± 1 °C) from 9:00 to 17:00 (8 h daily) and followed by a thermoneutral ambient temperature (23 ± 1 °C) from 17:00 to 09:00. Temperature elevation was achieved using an environmentally controlled chamber equipped with electric heating units and automated thermostatic regulation, with continuous monitoring via calibrated digital sensors to maintain target condition. Ambient temperatures in the experimental room were continuously monitored using data loggers (HOBO Pro Series, Model H08-032-08, ONSET Co., Cape Cod, MA, USA) to record both the high and low temperatures. Indoor relative humidity (RH) in the room was maintained at approximately 65% in accordance with standard management practices.

**Table 1:** Feed ingredients and nutrient content of the starter, grower, and finisher diets (kg/ton).

Ingredients	Starter (0–14 d)	Grower (15–28 d)	Finisher (29–42 d)
Corn grain	539	555	607
Soybean meal 48	382	359	306
Soybean oil	34.4	46.5	50.8
Limestone	15.40	13.90	12.80
Common salt	3.8	3.8	3.8
Vitamin premix^1^	1.00	1.00	1.00
Mineral premix^2^	1.00	1.00	1.00
DL-methionine	3.50	3.10	2.80
L-Lysin HCL	2.10	1.50	1.50
L-Threonine	1.40	1.00	0.80
Mono calcium phosphate	15.9	14.1	12.8
Choline CL-60%	0.030	–	–
Total	1000.0	1,000	1,000
Calculated nutrient, %			
Metabolic energy (ME), kcal/kg	3,000	3,100	3,200
Crude protein, %	23.0	21.15	20.0
Non phytate P, %	0.48	0.44	0.405
Calcium, %	0.96	0.87	0.81
D. Lysine, %	1.28	1.15	1.06
Sulfur amino acids, %	0.95	0.87	0.83
Threonine, %	0.86	0.77	0.71

^1^The vitamin-mineral premix components per kg: vit. A, 12000000 IU; vit. D3, 5,000,000 IU; vit. E, 80000 IU; vit. K3, 3,200 mg; vit. B1, 3,200 mg; vit. B_2_, 8,600 mg; vit. B_3_, 65,000 mg; pantothenic acid, 20,000 mg; vit. B_6_, 4,300 mg; biotin 220 mg; antioxidant (BHA+BHT), 50,000 mg; B_9_, 2,200 mg; B_12_, 17 mg; ^2^copper, 16,000 mg; iodine, 1,250 mg; iron, 20,000 mg; manganese, 120,000 mg; selenium, 300 mg, and zinc, 110,000 mg.

During the first week, the bulb was illuminated 24 h a day. From the second week until the end of the trial, the lighting schedule was adjusted to 23 h of light and 1 h of darkness (23L:1D). The average outside temperature during the experiment was approximately 26.4 °C, and the relative humidity varied between mild and moderate. All chicks were vaccinated against infectious bursal disease (IBD), Newcastle disease (ND), and infectious bronchitis (IB) according to standard protocols.

### 2.3 Stability test and storage conditions

Post-mixing probiotic viability was assessed by culture-based CFU enumeration. Representative feed samples were collected immediately after mixing and at predetermined storage intervals, serially diluted in sterile buffered peptone water, and plated on selective media appropriate for each microbial group, followed by incubation under recommended aerobic or microaerophilic conditions to determine viable counts. Feed was stored in sealed bags under standard storage conditions (dry, dark environment at approximately 22–25 °C and <65% relative humidity) for the duration of each feeding phase, and CFU recovery was expressed relative to initial counts to verify stability.

### 2.4 Proximate analysis of diet and bioactive compound analyses of RNFS

The proximate analysis of the macronutrients of the basic diet was determined according to the methods of AOAC (29). The probiotic RNFS was analyzed for bioactive compounds by gas chromatography–mass spectrometry (GC–MS; Agilent Technologies, CA, USA), with the percentages of compounds reported according to Azzam et al. (30).

### 2.5 Performance indices

Broilers were weighed individually on days 0, 14, 28, and 42 to assess average body weight. Feed intake was measured by calculating the difference between feed offered and feed residuals on the same days. Average daily feed intake (ADFI), average daily weight gain (ADWG), and FCR (ADFI/ADWG) were calculated for each replicate during the pre-stressed period (0–28), stressed period (29–42), and overall period (0–42 days).

### 2.6 Lymphoid organs development

Selected broilers (8 birds/treatment) were slaughtered at 42 days of age in accordance with halal guidelines and institutional animal care standards, emphasizing humane handling and rapid exsanguination. Birds were restrained and slaughtered by a trained operator using a sharp knife to sever the major neck vessels, ensuring prompt blood loss while minimizing distress. Procedures were conducted under hygienic conditions to maintain carcass quality and meat safety. The carcasses were eviscerated, processed, and weighed without neck, skin, abdominal fat, and internal organs. The lymphoid organs, including the bursa, spleen, and thymus, were excised and weighed. The weights of the lymphoid organs were expressed both as absolute values (g) and relative to the weight of the hot carcass, as stated by Alqhtani et al. (31).

### 2.7 Histopathology measurements

From each bird in the highest level of the RNFS group, a 2-cm ileal segment was collected without applying pressure on the tissue. Samples were fixed in 10% phosphate-buffered formalin for 72 h, dehydrated in graded alcohols, embedded in paraffin, sectioned at 5 μm thickness using a microtome, and stained with hematoxylin and eosin (H&E) as per Qaid et al. (32). Histological assessments were conducted under a light microscope at magnifications of 100×.

### 2.8 Blood biochemical analysis

On day 42, blood samples (*n* = 8 samples per treatment) were drawn from the wing vein and allowed to clot at room temperature for 2 h. Serum was separated by centrifugation at 3,000 rpm for 15 min at 30 °C, then stored at −20 °C until analysis (33). Biochemical markers assessed included alanine aminotransferase (ALT), aspartate aminotransferase (AST), urea, creatinine, glucose, cholesterol, total protein, and albumin. Globulin concentration was calculated as the difference between total protein and albumin. All parameters were measured using commercial diagnostic kits (Randox, London, UK) and a spectrophotometer (Randox, London, UK), based on absorbance readings in accordance with the manufacturer’s instructions.

### 2.9 Gene expression of immune response in small intestine

At 42 days of age, total RNA was extracted from ileal tissue using TRIzol™ Reagent (Invitrogen, Carlsbad, CA) according to the manufacturer’s instructions (34). For gene expression analysis, ileal tissues from two birds were pooled to constitute one biological replicate, yielding four pooled samples per treatment (*n* = 8 birds/treatment). Pooling was applied to reduce technical variance and tissue heterogeneity, ensure sufficient RNA yield, improve homogenization, and minimize biological noise arising from localized variability in mucosal gene expression. RNA concentration and purity were determined using a NanoDrop™ 2000 spectrophotometer (Thermo Fisher Scientific, Waltham, MA), and RNA integrity was confirmed by agarose gel electrophoresis. First-strand cDNA was synthesized from 1 μg of total RNA using the iScript™ cDNA Synthesis Kit (Bio-Rad Laboratories, Hercules, CA). Primers specific for *MUC-2, Claudin-1,* and *IgA* were designed based on published sequences and validated against the *Gallus gallus* nucleotide database at the National Center for Biotechnology Information (NCBI; https://www.ncbi.nlm.nih.gov/nuccore/), as presented in Table 2. Quantitative real-time PCR was performed using SYBR® Green Master Mix (Applied Biosystems, Foster City, CA) on a QuantStudio™ 3 Real-Time PCR System (Applied Biosystems). *β*-Actin was used as the internal reference gene, and relative gene expression levels were calculated using the 2^−ΔΔCt^ method.

**Table 2:** Primer sequences used for quantifying gene expression via real-time PCR.

Target gene	Gene reference sequence/accession number	Primer sequences (5′ → 3′)
HSP70	NM_001006685.1	F-CGGGCAAGTTTGACCTAA R-TTGGCTCCCACCCTATCTCT
sIgA	NM_205504 NM_205381	F: TCGAGGTGATCAACAAGCTG R: CTGCAGCAAAGTGAAGGACA
MUC-2	XM_013989745.1	F: GGTCATGCTGGAGCTGGACAGT R: TGCCTCCTCGGGGTCGTCAC
Claudin-1	NM_001013611.2	F-TGGCCACGTCATGGTATGG R -AACGGGTGTGAAAGGGTCATAG
Occludin	NM_205128.1	F-ACGGCAGCACCTACCTCAA R-GGGCGAAGAAGCAGATGAG
*β*-Actin	NM_205518.2	F-ATGGCTCCGGTATGTGCAAG R-CAACCATCACACCCTGATGTC

Yang et al. (34) and Criado-Mesas et al. (74). *β*-actin used as the internal reference gene for normalization.

### 2.10 Statistical analysis

All data were analyzed using one-way analysis of variance (ANOVA) within the General Linear Model (GLM) procedure of SAS software SAS (35); SAS Institute Inc., Cary, NC, USA. The statistical model used was as follows:


$$\gamma_{\mathrm{ij}}=\mu +T_{i}+e_{\mathrm{ij}}$$

Where *Y_ij_* is the individual observation, μ is the overall mean, *T_i_* is the fixed effect of the *i*th treatment, and e*_ij_* is the random residual error. Prior to statistical analysis, data normality was assessed using the Kolmogorov–Smirnov test. For growth performance parameters, the experimental unit was the pen (8 pens per treatment), while for other measured variables, individual birds were considered the experimental unit (*n* = 8 birds or samples per treatment). The statistical model accounted for the non-independence associated with pooled samples of genes expression by considering each pool as the experimental unit in the analysis. Treatment means were compared using Tukey’s Honestly Significant Difference (HSD) test at a significance level of *p* < 0.05. Data are presented as means ± standard error of the mean (SEM). The chosen sample size ensured adequate statistical power, exceeding the commonly accepted threshold of 80%. This level of power minimizes standard error and increases the likelihood of detecting true differences among treatments, if present.

## 3 Results

### 3.1 Proximate analysis of chemical composition of broilers diet

The analysis of the nutrient content of the basic diet is shown in Table 3. The moisture content was 71 g/kg (7.1%) in the grower diet and 79 g/kg (7.9%) in the finisher diet, indicating a slightly higher moisture content in the finisher phase. In contrast, the dry matter content was 929 g/kg (92.9%) in the grower feed and 921 g/kg (92.1%) in the finisher feed, providing a uniform dry matter basis for accurate nutrient comparison. The grower diet had a higher crude protein content (20.67% DM) than the finisher diet (18.24% DM). Conversely, the finisher diet had a higher starch (437 g/kg) and nitrogen-free extract (NFE; 65.04% DM) content, indicating a shift in energy requirements for final weight gain during the finisher phase. The fiber content was 29 g/kg (3.12% DM) in the grower diet compared to 26 g/kg (2.82% DM) in the finisher diet, and the ash content was 53 g/kg (5.71% DM) in the grower diet compared to 46 g/kg (4.99% DM) in the finisher diet. Overall, the composition of the basal diet fed to broilers in the grower and finisher phases met the expected Nutrition Specifications standards of ROSS 308.^1^

**Table 3:** Proximate analysis of chemical composition of broilers basal diet on a feed basis and dry matter basis^1^.

Item	Grower		Finisher	
	(g/kg; as-fed)	(%; on DM basis)	(g/kg; as-fed basis)	(%; as DM basis)
Moisture	71	7.1	79	7.9
Dry matter (DM)	929	92.9	921	92.1
Crude ash	53	5.71	46	4.99
Crude protein	192	20.67	168	18.24
Crude fat (E. E.)	80	8.61	81	8.79
Crude fiber	29	3.12	26	2.82
Starch	403	–	437	–
NFE^2^	575	61.89	599	65.04
Total	1,000	100	1,000	100

^1^The chemical composition analysis was performed in duplicate. ^2^NFE, nitrogen-free extract. NFE is represent the feed's soluble carbohydrates, such as starch and sugar, and is calculated by subtracting the original sample weight from the sum of the weights of moisture, ether extract, crude protein, crude fiber, and ash.

### 3.2 RNFS phytochemistry

The phytochemical composition of the RNFS probiotic was analyzed by GC–MS and revealed a complex profile of bioactive ingredients, as shown in Supplementary Table S1. A total of 48 bioactive compounds in RNFS were identified, consisting mainly of phenolic derivatives, organic acids, fatty acid esters, alcohols, and esters. Among the most important constituents were trimethyl 1,2,3-propanetricarboxylate (9.52%), phenol (9.17%), and 2,4-di-tert-butylphenol (6.86%). In addition, several methyl esters of fatty acids, such as methyl palmitate and methyl stearate were found.

### 3.3 Stability test and storage conditions

The multi-strain-multi-species probiotic solution used (RISCO–NUTRIFOUR®; RNFS) is manufactured by Al Raya Specialties Industrial Factory (Jeddah, Saudi Arabia). Post-mixing CFU enumeration of RISCO–NUTRIFOUR solution confirmed that probiotic viability remained stable throughout feed preparation and storage. Viable counts of all included microorganisms remained within the manufacturer’s specified ranges, with recovery rates exceeding 90% of the initial concentrations or colony-forming units (CFU) across the starter, grower, and finisher phases. Viable counts for RNFS were *Bacillus subtilis* (1 × 10^9^ CFU/mL), *Lactobacillus* species *harbinensis* and *Parabunchneri* (1 × 10^9^ CFU/mL each), *Rhodopseudomonas species palustris and shaeroides* (1 × 10^7^ CFU/mL each), *Candida ethanolic* (1 × 10^5^ CFU/mL), and *Saccharomyces cerevisiae* (1 × 10^5^ CFU/mL). No substantial declines in CFU counts were observed under the storage conditions applied (22–25 °C; <65% RH), indicating that the probiotic consortium-maintained stability and dose integrity during the feeding period.

### 3.4 Performance indices

The performance indices of the broilers over 42 days are shown in Table 4. RNFS supplementation had a statistically significant (*p* < 0.05) effect on the main broiler performance indices such as ABW, ADWG, and FCR. On day 42, the highest ABW value (*p* < 0.05) was observed in broilers receiving RFNS at a dosage of 0.1% (3,000 g), which was significantly higher than in the negative control (2,809 g) by 6.8%. This indicated that supplementation improved growth performance. ADWG during both the early (0–28 d) and late (29–42 d), as well as overall (0–42 d) phases were significantly increased by RNFS supplementation, particularly at 0.1%, with values 7.2, 6.3, and 6.4% higher than the negative control, respectively.

**Table 4:** Performance indices in broilers fed experimental diets supplemented with RISCO-NUTRIFOUR over 42 days.

Treatment	RISCO–NUTRIFOUR			0.1% *Bacillus subtilis*	0.1% *Saccharomyces cerevisiae*	Negative control	Standard error	*p-*value
Item	0.4%	0.2%	0.1%					
Average body weight (ABW; g/bird)								
ABW d 42	2935^ab^	2975^a^	3000^a^	2868^ab^	2872^ab^	2809^b^	46.9	0.045
Average weight gain (ADWG; g/bird/day)								
0–28 days	53.50^ab^	54.25^ab^	55.25^a^	52.95^b^	52.55^b^	51.55^c^	1.22	0.033
29–42 days	99.6^ab^	101.0^a^	101.8^a^	96.5^b^	97.6^ab^	95.8^b^	2.72	0.046
0–42 days	68.9^ab^	69.8^a^	70.1^a^	67.3^b^	67.4^b^	65.9^b^	1.12	0.048
Average feed intake (ADFI; g/bird/day)								
0–28 days	74.3	74.45	75.01	73.10	72.85	75.50	1.881	0.889
29–42 days	150.3	151.7	146.1	149.3	152.8	154.3	5.41	0.918
0–42 days	99.6	100.8	99.3	97.9	99.7	101.3	2.57	0.760
Feed conversion ratio (FCR; g: g)								
0–28 days	1.39^b^	1.37^bc^	1.36^c^	1.38^b^	1.39^b^	1.46^a^	0.029	0.048
29–42 days	1.52^b^	1.50^b^	1.45^c^	1.55 ^ab^	1.57^ab^	1.61^a^	0.059	0.046
0–42 days	1.45 ^b^	1.44^b^	1.42^b^	1.45^b^	1.48^ab^	1.54^a^	0.032	0.050

*n* = 8 cage/treatment (6 birds for each replicate). ^a–c^Means with different superscripts on the same line differ considerably (*p* < 0.05).

There were no significant differences in ADFI (*p* > 0.05). Lower FCR scores indicate improved feed efficiency, with the 0.1% RNFS supplement having the best efficiency (1.36 for d 0–28 and 1.45 for d 29–42 and 1.42 for d 0–42 overall, representing improvements of 10, 16, and 12 points, respectively). The control group had the worst FCR scores, indicating that the probiotics improved nutrient utilization. ADWG and FCR in the RFNS groups were comparable to, or better than, those of the commercial single-probiotic positive control groups, particularly at the 0.1% inclusion level. Even the highest dose of RNFS (0.4%) led to a numerically higher ADWG and a significantly improved FCR compared to the negative control group.

### 3.5 Lymphoid organs development

The absolute (g) and relative weights (% hot carcass weight) of lymphoid organs from broilers fed RNFS and other probiotic treatments, measured at day 42, are shown in Table 5. No significant differences (*p* > 0.05) were observed in the absolute or relative weights of the spleen and bursa between the treatment groups compared to the negative control group. Thymus weight, both absolute (*p* = 0.051) and relative (*p* = 0.054), approached statistical significance, indicating a potential trend towards reduced thymus development in broilers supplemented with lower levels of RNFS, particularly at 0.1%.

**Table 5:** Lymphoid organs development of the stressed broilers supplemented RISCO-NUTRIFOUR in feed base measured at day 42.

Treatment	RISCO–NUTRIFOUR			0.1% *Bacillus subtilis*	0.1% *Saccharomyces cerevisiae*	Negative control	Standard error	*p*-value
Item	0.4%	0.2%	0.1%					
Absolute weight (g)								
Spleen	2.83	2.34	2.09	2.10	2.24	2.37	0.246	0.307
Thymus	9.69	9.44	6.69	7.83	8.77	11.05	0.864	0.051
Bursa	3.92	4.46	4.46	4.70	4.17	4.03	0.418	0.760
Relative weight (g/100 g carcass weight)								
Spleen	0.146	0.122	0.115	0.117	0.117	0.121	0.011	0.402
Thymus	0.516	0.492	0.369	0.434	0.460	0.530	0.045	0.054
Bursa	0.209	0.232	0.247	0.259	0.218	0.206	0.022	0.484

*n*= 8 samples/treatment.

### 3.6 Quantitative and qualitative histomorphometry evaluation

Quantitative ileal histomorphometry at day 42 showed significant treatment effects on villus architecture but not on crypt depth–related indices (Table 6). Villus height differed among groups (*p* = 0.0003), with the highest value observed in broilers receiving 0.1% RNFS (593 μm), which was significantly greater than the negative control (560.6 μm) and the 0.4% RNFS group, while intermediate values were noted for 0.2% RNFS and the single-species probiotic groups. Villus surface area (VSA) followed a similar pattern (*p* = 0.001), peaking at 0.1% RNFS (0.328 mm^2^) and being lowest in the negative control (0.303 mm^2^). In contrast, crypt depth did not differ significantly among treatments (*p* = 0.267). Consequently, the villus height-to-crypt depth ratio (VH/CD) showed no statistical variation (*p* = 0.103), although numerically higher values were recorded in RNFS-supplemented groups, particularly at 0.1% inclusion. Overall, RNFS, especially at 0.1%, improved villus structural development without altering crypt morphology under heat stress.

**Table 6:** Quantitative histomorphometry parameters of the stressed broilers supplemented RISCO-NUTRIFOUR in feed base measured at day 42.

Treatment	RISCO–NUTRIFOUR			0.1% *Bacillus subtilis*	0.1% *Saccharomyces cerevisiae*	Negative control	Standard error	*p-*value
Item	0.40%	0.20%	0.10%					
Villus height (μm)	566.6^bc^	576^abc^	593^a^	583.6^ab^	572.6^bc^	560.6^c^	4.360	0.0003
VSA, mm^2^	0.309^bc^	0.315^abc^	0.328^a^	0.320^ab^	0.312^bc^	0.303^c^	0.004	0.001
Crypt depth (μm)	173.8	174.2	176	174.4	173.6	172.2	1.052	0.267
VH/CD	3.26	3.31	3.37	3.35	3.30	3.26	0.032	0.103

VSA, villus surface area; VH/CD, villus height/crypt depth.

**Figure 1:**
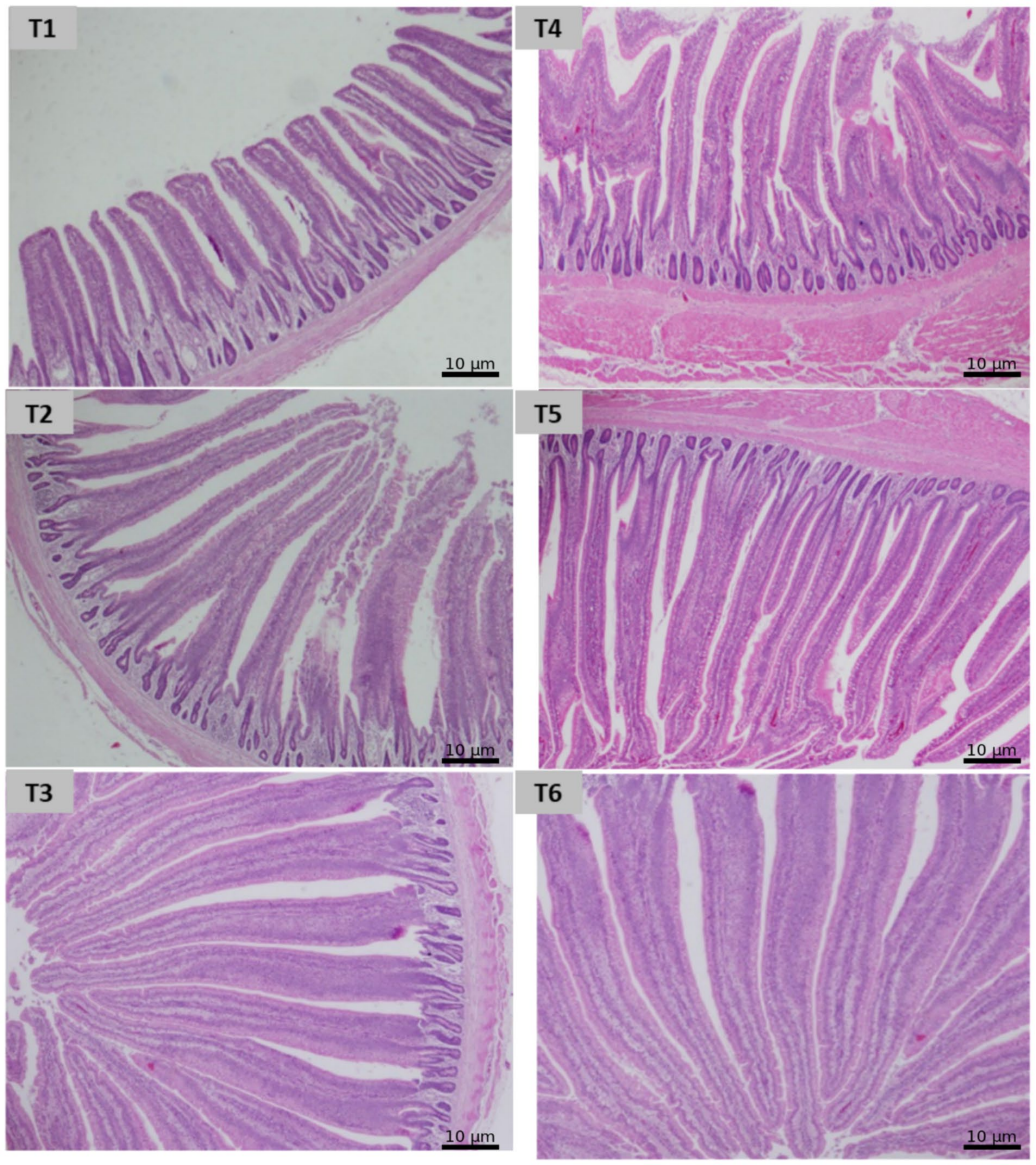
Histological qualitative evaluation of ileal sections at day 42 stained with hematoxylin and eosin revealed preserved intestinal architecture across all treatments. The histological findings indicate that RNFS supplementation at all tested doses (T1–T3 at 0.1, 0.2%, or 0.4%) maintained ileal structural integrity and mucosal health under heat stress conditions, with morphology comparable to probiotic controls (T4: 0.1% *Saccharomyces cerevisiae* and T5: 0.1% *Bacillus subtilis*) and negative control (T6) without detectable tissue injury. (Magnification = 100X and scalebar = 10 *μm* for all images).

As shown in Figure 1, histological qualitative evaluation of ileal sections at day 42 revealed preserved intestinal architecture across all treatments. In broilers receiving RNFS at 0.1, 0.2, and 0.4%, the mucosa exhibited well-organized, finger-like villi with intact epithelial lining and clearly defined brush borders. Villus height and morphology appeared uniform, with no evidence of epithelial erosion, villus fusion, or tip necrosis. The lamina propria showed normal cellularity without edema, hemorrhage, or excessive inflammatory cell infiltration. Crypts of Lieberkühn were distinct and regularly distributed, with no signs of hyperplasia or degeneration.

Similarly, tissues from the *Bacillus subtilis* and *Saccharomyces cerevisiae* groups displayed normal mucosal integrity, comparable to RNFS treatments. The muscularis mucosae and submucosa remained structurally intact, and no vascular congestion or tissue disorganization was evident. In the negative control under heat stress, the histomorphologies of villi were still preserved, though villi appeared slightly less robust and slenderer, suggesting mild stress-related morphological modulation rather than pathology. Notably, no lesions, ulceration, goblet cell depletion, or inflammatory damage were observed in any group.

Overall, the histological findings indicate that RNFS supplementation at all tested doses maintained ileal structural integrity and mucosal health under heat stress conditions, with morphology comparable to probiotic controls and without detectable tissue injury.

### 3.7 Serum biochemical profile

The biochemical profile of broilers fed with RNFS and other probiotic treatments, measured at day 42 is presented in Table 7. No significant differences (*p* > 0.05) were observed among the stressed broiler groups in serum albumin, globulin, total protein, creatinine, urea, or ALT levels. Significant reductions in triglyceride and cholesterol levels in the groups treated with RNFS (especially 0.2% RNFS) and individual probiotics indicate improved lipid metabolism, with RNFS at 0.2 and 0.1% and *Saccharomyces* groups showing the lowest cholesterol levels. AST activity was significantly lower in all RNFS and probiotic groups compared to the control group. Although a trend towards lower ALT values in the RNFS groups is not statistically significant (*p* = 0.052), it could indicate a possible benefit for liver function.

**Table 7:** Biochemical profile of the stressed broilers supplemented RISCO–NUTRIFOUR in feed base measured at day 42.

Treatment Item	RISCO–NUTRIFOUR			0.1% *Bacillus subtilis*	0.1% *Saccharomyces cerevisiae*	Negative control	Standard error	*p*-value
	0.4%	0.2%	0.1%					
Blood proteins								
Albumin (g/dL)	1.49	1.50	1.53	1.59	1.64	1.08	0.354	0.896
Globulin (g/dL)	2.76	3.43	3.60	3.43	3.26	3.03	0.707	0.963
Total protein (g/dL)	4.25	4.93	5.13	5.02	4.90	4.11	1.06	0.973
Lipid profile								
Triglyceride (mg/dL)	92^e^	91^e^	112^d^	174^b^	138^c^	193^a^	2.83	<0.0001
Cholesterol (mg/dL)	177^ab^	148^c^	150^c^	170^b^	139^c^	183^a^	2.89	<0.0001
Renal function								
Creatinine (mg/dL)	1.76	1.74	1.66	1.67	1.69	1.71	0.454	0.99
Urea (mg/dL)	43.9	43.6	41.1	43.7	43.5	43.0	2.41	0.758
Hepatic function								
AST (mL)	57.3^ab^	54.6^b^	53.8^b^	54.6^b^	59.1^ab^	63.1^a^	2.41	0.001
ALT (mL)	15.2	15.7	14.6	19.1	19.4	19.3	1.35	0.052

*n* = 8 samples/treatment. ^a–e^Means with different superscripts on the same line differ considerably (*p* < 0.05).

### 3.8 Gene expression of immune response in small intestine

**Figure 2:**
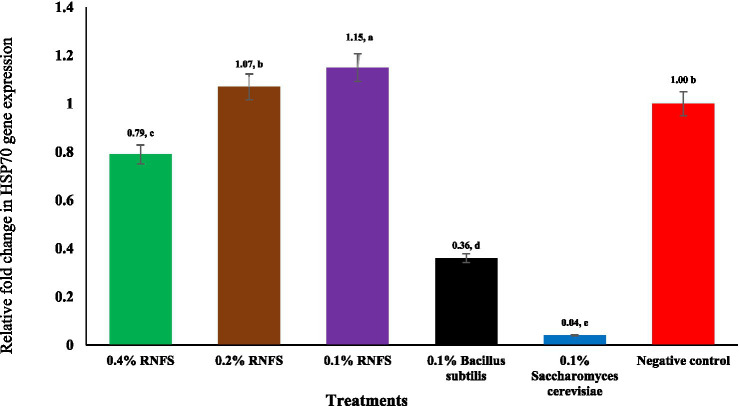
Effect of RISCO–NUTRIFOUR (RNFS) supplementation on heat shock protein 70 (HSP70) gene expression in ileal segment of broiler chickens under heat stress measured at day 42. Data are expressed as fold-change relative to the negative control (set as 1; mean ± 0.016. Eight birds were pooled into four replicates (*n* = 4 replicates). ^a-e^ Different superscript letters indicate significant differences between treatments (*p* < 0.05).

**Figure 3:**
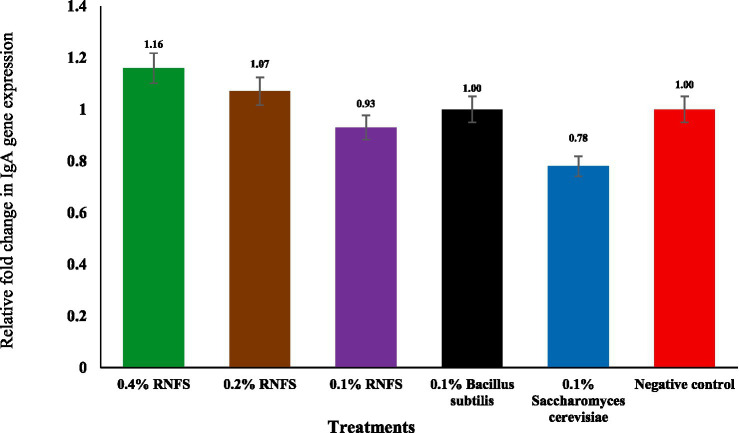
Effect of RISCO–NUTRIFOUR (RNFS) supplementation on immunoglobulin A (IgA) gene expression of immune response in ileal segment of broiler chickens under heat stress measured at day 42. Data are expressed as fold-change relative to the negative control (set as 1) and presented as mean ± 0.318 and *p* = 0.937. *n* = 4 pooled replicates (total = 8 birds).

**Figure 4:**
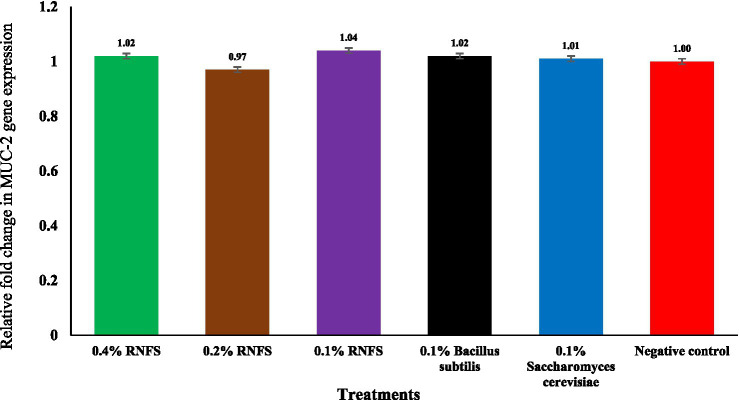
Effect of RISCO–NUTRIFOUR (RNFS) supplementation on mucin-2 (MUC-2) gene expression of immune response in ileal segment of broiler chickens under heat stress measured at day 42. Data are expressed as fold-change relative to the negative control (set as 1) and presented as mean ± 0.396 and *p* = 0.99. Eight birds were pooled into four replicates (*n* = 4 replicates).

**Figure 5:**
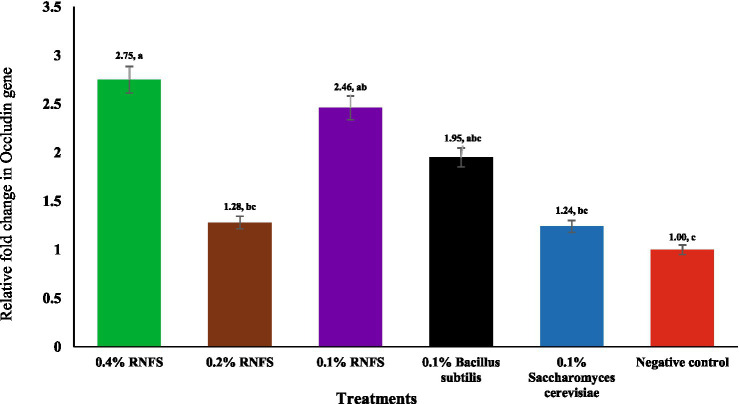
Effect of RISCO–NUTRIFOUR (RNFS) supplementation on occludin gene expression of immune response in ileal segment of broiler chickens under heat stress measured at day 42. Data are expressed as fold-change relative to the negative control (set as 1) and presented as mean ± 0.408. *n* = 4 pooled replicates (total = 8 birds). ^a-c^Different superscript letters indicate significant differences between treatments (*p* = 0.034).

**Figure 6:**
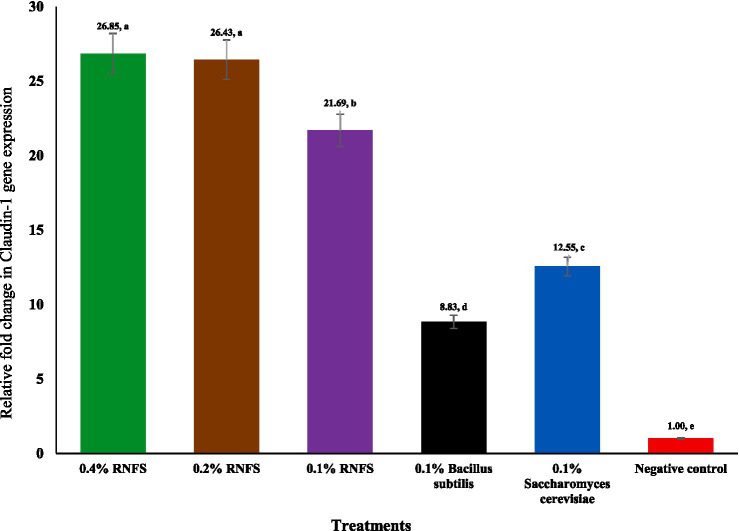
Effect of RISCO–NUTRIFOUR (RNFS) supplementation on claudin-1 gene expression of immune response in ileal segment of broiler chickens under heat stress measured at day 42. Data are expressed as fold-change relative to the negative control (set as 1) and presented as mean ± 0.608. *n* = 4 pooled replicates (total = 8 birds). ^a-e^Different superscript letters indicate significant differences between treatments (*p* <0.0001).

Figures 2–6 illustrate that dietary RNFS supplementation under heat stress reduced HSP70 expression while upregulating IgA, MUC-2, occludin, and claudin-1 gene expression. HSP70 expression was significantly reduced in probiotic-treated groups compared with the negative control (*p* < 0.05), with the greatest suppression observed in the single-species probiotic groups; RNFS at 0.4% also decreased stress-gene expression relative to lower inclusion levels (Figure 2). IgA and MUC-2 expression showed only minor, non-significant variation among treatments (*p* > 0.05; Figures 3, 4). In contrast, tight junction–related genes were strongly responsive to RNFS. Occludin expression was significantly upregulated, particularly at 0.4 and 0.1% RNFS, representing increases of 175 and 146% over the negative control, respectively, indicating enhanced epithelial barrier integrity compared with the control and most single-species treatments (Figure 5).

Similarly, claudin-1 expression was substantially elevated in RNFS groups, with the highest responses at 0.4 and 0.2%, which reached values of 26.85 and 26.43, representing an increase of 2.59 and 2.54% over the negative control, supporting improved tight junction function under heat stress (Figure 6). Overall, RNFS supplementation under heat stress primarily strengthened intestinal barrier-associated gene expression, while modulation of stress and mucosal immune markers showed dose- and probiotic-type–dependent variation.

## 4 Discussion

Broiler chickens are highly susceptible to various stressors, including thermal, immunological, and oxidative stress, which together affect intestinal mucosal function, immune-competence, and overall productivity (23, 36). The current study shows that dietary supplementation with RNFS, a probiotic solution combining multiple microbial strains-species and bioactive phytochemicals, mitigates the adverse effects of HS by improving growth performance, modulating immune system gene expression and enhancing gut barrier function. These effects are consistent with the growing body of evidence supporting the role of natural feed additives such as yeast, probiotics and antioxidant phytochemicals in alleviating stress and maintaining gut health (17, 37, 38). In particular, RNFS enhanced the expression of tight junction (TJ) proteins such as occludin and claudin-1, which are essential components of the epithelial barrier and regulate intestinal permeability (6, 7). Damage to these proteins is known to disrupt the integrity of the barrier and allow the translocation of pathogens and endotoxins that exacerbate systemic inflammation and disease (8, 39). By significantly upregulating these TJ markers, especially in 0.4% RNFS, this study confirms the ability of RNFS to restore the integrity of the mucosal barrier under HS.

Oxidative stress, usually triggered by environmental conditions such as heat, poor feed quality and dense housing, is an important factor in TJ disruption and mucosal injury (40, 41). The antioxidant-rich composition of RNFS, including phenolic compounds and long-chain fatty acid (LCFA) esters, likely contributes to the observed reduction in HSP70 expression and protection of intestinal structure. These findings are consistent with those of Lee et al. (42), who reported that dietary antioxidants preserve TJ integrity by reducing ROS-induced cellular damage.

Furthermore, the stress-reducing effects of RNFS were not only evident at the molecular level but were also reflected in the general physiological state of the birds. Although the ratio of heterophils to lymphocytes (H/L) was not directly measured in this study, the immunomodulatory effect derived from IgA upregulation is consistent with the results of Alaqil et al. (43), where *Lactobacillus acidophilus* reduced the H/L ratio, indicating a lower systemic burden. The role of *Lactobacillus* species in RNFS, such as *Lactobacillus parabuchneri* and *Lactobacillus harbinensis*, could similarly contribute to stress reduction and immunomodulation.

The detected enhancements in growth parameters and FCR, especially at a dosage of 0.1%, are consistent with several studies demonstrating the benefits of probiotic supplementation in poultry. For example, Zhang et al. (44) and Soumeh et al. (45) reported that probiotic mixtures improve nutrient utilization, resulting in better body weight gain and FCR, likely due to improved gut microbiota balance and enzymatic activity. There were no significant differences in ADFI, ensuring that significant differences were adequately identified, suggesting that the improvement in growth performance was not due to increased feed consumption but to better feed efficiency.

Interestingly, the current results showed a dose-dependent bifurcation of the RNFS effects: lower doses (especially 0.1%) were more effective in promoting growth performance, while higher doses (0.4%) produced better immunomodulatory results, such as increased expression of IgA, occludin and claudin-1 and decreased expression of HSP70. This pattern is consistent with the findings of Guo *et al* (46), who reported that low-dose probiotics increased ADWG by 1.14-fold and 1.27-fold compared with the medium-and high-dose groups, while Liu et al. (47) reported that higher doses better supported immune and intestinal barrier functions, possibly through greater stimulation of mucosal immunity. In contrast to Hossain et al. (48), who found that feeding 500 mg lyophilized (freeze-dried) probiotics/kg feed outperformed feeding 250 mg lyophilized probiotics/kg feed in terms of ADWG. Similarly, Liu et al. (49) found that broilers in the high (5 × 10^7^ CFU/mL) *Lactiplantibacillus plantarum* HW1 group had the highest ADWG during days 22 to 42 and 1 to 42 compared with the medium, low, and control groups. In terms of FCR, high probiotic supplementation during days 22 to 42 was similar to medium and low probiotic supplementation and decreased FCR compared to the control (49). In the current study, crude fat levels in the basal diets were stable (approximately 8.6–8.8% DM), contributing to the energy density required under HS conditions. The relatively low and stable crude fiber content (<3.2% DM) supports digestibility, while the ash content was slightly higher in the grower phase, possibly due to mineral supplementation to support skeletal development.

Our results show that the treatments had no effect on the absolute or relative weight of the broilers’ lymphoid organs after the birds had been fed RNFS for 42 days. These results are consistent with several studies in which different strains of probiotics were administered for different periods of time and by different routes with no effect on lymphoid organ weight (45, 50, 51).

The significant reduction in HSP70 gene expression under RNFS, *Bacillus subtilis*, and *Saccharomyces cerevisiae* treatments confirms the ability of probiotics to mitigate cellular stress responses. These results support previous reports by Aydin and Hatipoglu (52) and Khan et al. (53), who observed similar downregulation of HSP70 in probiotic-treated chickens under HS, suggesting improved thermotolerance through reduced oxidative and metabolic stress. In addition, the upregulation of occludin and claudin-1 of TJ proteins under RNFS treatment is consistent with studies by Hernández-Coronado et al. (54) and Song et al. (55), suggesting that probiotics strengthen intestinal barrier function and attenuate HS-induced intestinal permeability. The particularly strong increase in claudin-1 expression in the RNFS group (21.69- to 26.85-fold) compared to the negative control and the live yeast (12.55-fold) and *Bacillus subtilis* (8.63-fold) groups exceeds commonly reported levels, possibly due to the synergistic effect of the phytochemical constituents of RNFS, including phenolic compounds and fatty acid esters, which are known to support membrane integrity and anti-inflammatory responses.

Although the expression of mucin-2 (MUC-2) showed only slight fluctuations, this does not contradict earlier studies. For example, Mohammed et al. (56) found that the effects of probiotics on MUC-2 are context-dependent and may be due to changes in microbial ecology or TJ modulation. Ileal histomorphology corroborated the molecular findings, as birds supplemented with RNFS at all inclusion levels maintained intact mucosal architecture under heat stress. The preservation of villus structure, epithelial continuity, and crypt organization, together with the absence of inflammatory infiltration or tissue damage, indicates sustained barrier integrity and epithelial turnover. This structural stability aligns with the upregulation of mucin-2, occludin, and IgA expression and supports the role of RNFS in reinforcing gut barrier function and mucosal resilience during thermal stress.

The divergent effects on growth performance and gut molecular responses indicate a dose-dependent resource allocation trade-off under heat stress. Lower RNFS inclusion (0.1%) likely enhances digestive efficiency and nutrient utilization with minimal metabolic cost, favoring growth performance, whereas higher inclusion (0.4%) preferentially strengthens mucosal immunity and intestinal barrier integrity, as reflected by increased IgA, mucin-2, and occludin expression and reduced HSP70. These responses likely arise from the combined action of the probiotic consortium and bioactive phytochemicals, including phenolic derivatives and fatty acid esters with reported antioxidant and immunomodulatory activity. Collectively, the findings suggest that lower RNFS doses support production efficiency, while higher doses prioritize intestinal protection under heat stress. However, targeted analyses of oxidative stress and inflammatory signaling markers are required to confirm this hypothesis.

Biochemically, RNFS decreased serum triglycerides and cholesterol levels, which is consistent with the lipid-lowering effects documented for probiotics with fermentative or enzymatic capabilities (57). AST activity was significantly lower in all RNFS and probiotic groups compared to the control group, indicating hepatoprotective effects under HS conditions. The absence of adverse changes in liver and renal function markers also supports the safety and tolerability of RNFS and is consistent with the results of previous safety evaluations of *Bacillus*-based probiotics in poultry (58, 59).

Probiotics support immunomodulation by reducing inflammation and redirecting energy from immune responses to growth (60–63). The observed diverse effects of the RNFS probiotic can be attributed to its diverse microbial consortium, which includes *Bacillus subtilis*, *Saccharomyces cerevisiae*, *Lactobacillus* spp., *Rhodopseudomonas* strains, and *Candida ethanolic*. *Bacillus subtilis*, a proven probiotic, contributes to nutrient digestion and antimicrobial peptide production, which is consistent with the observed improved FCR. *Saccharomyces cerevisiae* and *Candida* species have been shown to modulate lipid metabolism and host antioxidant defenses (63, 64), consistent with the reduction in serum triglycerides and HSP70 expression. *Lactobacillus parabuchneri* and *Lactobacillus harbinensis* are novel strains known to improve mucosal immunity and TJ integrity (65), which likely supports the upregulation of IgA, occludin and claudin-1 observed in this study. The inclusion of *Rhodopseudomonas* spp.—photosynthetic bacteria with antioxidant and immunostimulatory properties [24]- could be another explanation for the robust gut barrier responses, especially under HS. *Saccharomyces cerevisiae* constituents (*β*-glucans, mannans) activate innate immunity via macrophages and dendritic cells, while *Bacillus subtilis* and *Lactobacillus acidophilus* enhance mucosal immunity by improving barrier function and increasing IgA and anti-inflammatory cytokines (66). The modulation of the microbiota induced by probiotics also reduces oxidative stress and IL-6 levels, which supports muscle building (61, 62). SCFAs from fiber fermentation by *Lactobacillus* and *Bacillus* improve gut integrity and energy supply to the epithelium (67). In addition, *Saccharomyces cerevisiae* cell wall polysaccharides and metabolites (e.g., B-vitamins, amino acids) promote growth and carcass quality. Its ability to bind mycotoxins increases the performance of broilers, with the effective amount being 2.5–3.75 g/kg feed (68). This broad-spectrum microbial synergy could thus be responsible for the superior performance of RNFS as multi-strain and multi-species compared to single-strain probiotics.

In proximate analysis, the crude protein content in the grower diet was a higher than in the finisher diet, which is related to the increased protein requirements for muscle development in the early growth phase and the typical decrease in protein requirements as the birds mature. The grower diet was richer in protein to support early growth, while the finisher diet had a higher proportion of energy-yielding components such as starch and NFE. The nutrient profile was balanced and suitable for evaluating the effects of feed additives under HS conditions. The GC–MS profile of the probiotic RNFS mixture shows a rich diversity of bioactive phytochemicals with multiple biological functions that may collectively contribute to its efficacy as a dietary supplement under HS conditions in poultry. The detection of trimethyl 1,2,3-propanetricarboxylate (9.52%), a known anti-inflammatory and antioxidant agent (69), alongside phenol (9.17%), a potent antimicrobial and antiseptic compound (70), emphasizes the potential of RNFS to modulate oxidative damage and microbial balance in the gut. 2,4-Di-tert-butylphenol (6.86%), a lipid-soluble phenol (71), is particularly effective in neutralizing free radicals and thus supports cell protection during HS. The identification of methyl esters of LCFA, such as methyl palmitate and methyl stearate (72), underpins the ability of RNFS to improve cell membrane stability and promote energy metabolism, which is critical under HS conditions. These fatty acids also exhibit mild antimicrobial activity that contributes to the maintenance of intestinal integrity. In addition, compounds such as 1H-pyrazole, anisole, allyl alcohol and acetoin may contribute to synergistic antimicrobial activity and improved intestinal function (73). The variety of esters and alcohols detected indicates a formulation that is both functionally active and microbiologically stable. The identified chemical constituents indicate that RNFS enhances broiler performance and heat stress tolerance through antioxidant, anti-inflammatory, and gut microbiota–modulating mechanisms, providing a phytochemical basis for its efficacy. These actions promote epithelial homeostasis and resistance to HS, reflecting known effects of individual probiotic strains and plant bioactives, but are further enhanced by the synergistic formulation of RNFS.

Key limitations include the lack of direct gut microbiota profiling-microbial effects were inferred from host molecular markers-restricting confirmation of probiotic colonization and community shifts; the short, finisher-only heat stress model; the absence of an RNFS group under thermoneutral conditions; and the lack of a conventional anti-heat stress positive control. Future studies should incorporate microbiome and metabolomic analyses, earlier and prolonged heat exposure models, thermoneutral probiotic groups, and direct comparisons with established mitigation additives. Overall, the study extends current knowledge by highlighting the dose-dependent dual role of RNFS in supporting productivity and gut health under heat stress and emphasizing the need for precise dose optimization in probiotic feeding strategies.

## 5 Conclusion

In conclusion, the RNFS, multi-strain and multi-species probiotic, is effective in improving broiler performance under HS conditions, especially at the 0.1% level, as well as the expression of immunity genes and intestinal barrier function, especially at the 0.4% level. Additionally, the preservation of ileal histoarchitecture across RNFS-supplemented groups confirms its role in maintaining intestinal structural integrity under heat stress conditions. Supplementation with RFNS at 0.1% resulted in remarkable improvements in ABW by 6.8% at day 42 and improvements in ADWG by 7.2, 6.3, and 6.4% and FCR by 10, 16, and 12 points during the 0–28, 29–42, and 0–42 periods, respectively, compared to the negative control group. Supplementation with RFNS at 0.4% resulted in remarkable improvements in several markers of gut health: a 16% increase in IgA gene expression, a 175% increase in occludin expression, an exceptional 2,585% increase in claudin-1 expression, and a reduction in HSP70 gene expression compared to the negative control group. The observed improvements in FCR, IgA, occludin and claudin-1 expression - in conjunction with a significant reduction in HSP70 - demonstrate that resilience to HS can be promoted without compromising lymphoid organ development or metabolic function. The phytochemical complexity of RNFS underpins its antioxidant and immunomodulatory activities. Although RNFS showed promising biological effects under experimental heat stress conditions, practical adoption in commercial poultry systems requires further evaluation of economic feasibility, storage stability, and compatibility with standard feed manufacturing practices.

## Data Availability

The original contributions presented in the study are included in the article/Supplementary material, further inquiries can be directed to the corresponding authors.

## Footnotes

1
https://aviagen.com
