# Supplementary material for: Synergistic effects of dietary RISCO-NUTRIFOUR probiotic on broiler performance, upregulation of intestinal immunoglobulin A and mucin-2 genes, enhancement of occludin expression, and downregulation of HSP70 under heat stress
Source: Front Vet Sci. 2026 Apr 22;13:1776015. doi: 10.3389/fvets.2026.1776015 (PMC13143784; doi:10.3389/fvets.2026.1776015)
Supplement: Supplementary file 1 [file Table_1.docx]

# **Supplementary Tables**

**Supplementary Table S1.** RISCO–NUTRIFOUR outcomes using GC–MS.

| **RT (min)** | **Area** | **Hit Name** | **%** | **MW** |
| --- | --- | --- | --- | --- |
| 18.576 | 3003457 | Trimethyl 1,2,3-propanetricarboxylate | 9.52 | 218.079 |
| 8.122 | 2892759 | Phenol | 9.17 | 94.042 |
| 20.631 | 2163619 | 2,4-Di-tert-butylphenol | 6.86 | 206.167 |
| 13.678 | 1735143 | Naphthalene, 2,3,6-trimethyl- | 5.50 | 170.11 |
| 12.746 | 1577819 | Silane, butyltrimethyl- | 5.00 | 130.118 |
| 31.125 | 1574963 | 11-Octadecenoic acid, methyl ester | 4.99 | 296.272 |
| 12.419 | 1445307 | 1H-Pyrazole, 1,5-bis- | 4.58 | 212.117 |
| 28.344 | 1310078 | Hexadecanoic acid, methyl ester | 4.15 | 270.256 |
| 9.518 | 1193730 | Butanedioic acid, dimethyl ester | 3.79 | 146.058 |
| 31.966 | 1104075 | 1-Octadecanol | 3.50 | 342.332 |
| 16.751 | 950612 | 2-Pentenedioic acid, 2-methoxy-, dimethyl ester | 3.01 | 188.068 |
| 6.652 | 730189 | Anisole | 2.32 | 108.058 |
| 3.985 | 651549 | Allyl alcohol | 2.07 | 130.081 |
| 20.178 | 622080 | trans-1,2-ethylene | 1.97 | 172.11 |
| 37.167 | 604587 | Bis (2-ethylhexyl) phthalate | 1.92 | 390.277 |
| 31.531 | 561298 | Methyl stearate | 1.78 | 298.287 |
| 30.261 | 556769 | Palmitic acid | 1.77 | 328.28 |
| 5.21 | 555794 | Isobutanol | 1.76 | 146.113 |
| 35.284 | 552144 | Hexanedioic acid, bis (2-ethylhexyl) ester | 1.75 | 370.308 |
| 33.236 | 534082 | Stearic acid | 1.69 | 356.311 |
| 13.793 | 489799 | Propylene glycol | 1.55 | 220.131 |
| 27.989 | 473636 | 7,9-Di-tert-butyl-1-oxaspiro (4,5) deca-6, 9-diene-2, 8-dione | 1.50 | 276.173 |
| 22.576 | 402060 | Ethoxyacetic acid | 1.28 | 176.087 |
| 29.477 | 395841 | 3-Eicosene, (E)- | 1.26 | 280.313 |
| 7.138 | 395053 | Ethanimidic acid | 1.25 | 203.116 |
| 5.822 | 384120 | Acetoin | 1.22 | 160.092 |
| 15.589 | 329973 | Benzenepropanoic acid, methyl ester | 1.05 | 164.084 |
| 31.016 | 329904 | 9,12-Octadecadienoic acid (Z, Z)-, methyl ester | 1.05 | 294.256 |
| 26.101 | 315598 | 1-Octadecene | 1.00 | 252.282 |
| 27.234 | 309789 | Phthalic acid, isobutyl nonyl ester | 0.98 | 348.23 |
| 28.401 | 298270 | Benzenepropanoic acid, 3,5-bis (1,1-dimethylethyl) -4-hydroxy-, methyl ester | 0.95 | 292.204 |
| 17.724 | 259676 | Benzenepropanoic acid, alpha.-hydroxy-, methyl ester | 0.82 | 180.079 |
| 32.567 | 257127 | 1-Hexacosene | 0.82 | 364.407 |
| 8.981 | 239941 | Benzohydroxamic acid | 0.76 | 281.127 |
| 22.284 | 220409 | Benzoic acid, 3,4-dimethoxy-, methyl ester | 0.70 | 196.074 |
| 5.416 | 220318 | 2-Pentanol | 0.70 | 160.128 |
| 9.959 | 208307 | Phenmetrazine | 0.66 | 249.155 |
| 14.256 | 194069 | Dimethyl 3-hydroxy-3-methylpentane-1,5-dioate | 0.62 | 190.084 |
| 13.478 | 187185 | Lactic acid | 0.59 | 234.111 |
| 17.501 | 180781 | Nonanoic acid | 0.57 | 230.17 |
| 7.521 | 175576 | 2-Pentenal, 2-methyl- | 0.56 | 98.073 |
| 15.818 | 159708 | 1,3-Bis-propene | 0.51 | 186.126 |
| 16.288 | 154326 | Catechol | 0.49 | 338.21 |
| 8.368 | 139997 | Methoxyacetic acid | 0.44 | 204.118 |
| 16.997 | 139387 | Tartronic acid | 0.44 | 336.124 |
| 14.891 | 119191 | Ethylene glycol | 0.38 | 206.116 |
| 11.641 | 117149 | 4-Hydroxybutanoic acid | 0.37 | 248.126 |
| 24.075 | 115583 | Adipic acid, butyl hexyl ester | 0.37 | 286.214 |
| **Total** | | | 100.00 |  |
